# Exploring Job-Related Factors and Exercise Intentions in Relation to Overall Physical Activity and Its Subdivisions

**DOI:** 10.3390/bs14100912

**Published:** 2024-10-08

**Authors:** Wei-Hsun Wang, Wei-Ting Hsu, Hsin-I Cheng, Ren-Hau Li, Shu-Ling Huang, Feng-Cheng Tang

**Affiliations:** 1 Department of Post-Baccalaureate Medicine, College of Medicine, National Chung Hsing University, Taichung 402, Taiwan; 132072@cch.org.tw; 2 Department of Golden-Ager Industry Management, Chaoyang University of Technology, Taichung 413, Taiwan; 3 Department of Orthopedics, Changhua Christian Hospital, Changhua 500, Taiwan; 4 Department of Construction Engineering, Chaoyang University of Technology, Taichung 413, Taiwan; wthsu@cyut.edu.tw; 5 Department of Leisure Services Management, Chaoyang University of Technology, Taichung 413, Taiwan; 6 Child Development Center, Far Eastern Memorial Hospital, New Taipei 220, Taiwan; 410283030@gms.ndhu.edu.tw; 7 Department of Psychology, Chung Shan Medical University, Taichung 402, Taiwan; davidrh@csmu.edu.tw; 8 Room of Clinical Psychology, Chung Shan Medical University Hospital, Taichung 402, Taiwan; 9 Department of Occupational Medicine, Changhua Christian Hospital, Changhua 500, Taiwan; 10 Graduate Institute of Clinical Medicine, National Chung Hsing University, Taichung 402, Taiwan

**Keywords:** physical activity, workplace health promotion, exercise intention

## Abstract

This study examined the relationships between job-related factors and overall physical activity (PA), including its subdivisions: leisure-time PA, transportation PA, and work-related PA. Additionally, this study investigated the associations between exercise intentions and different types of PA. A cross-sectional design was employed, utilizing a questionnaire to collect data on participants’ demographics, job-related characteristics, exercise intentions, and levels of PA. A total of 400 full-time workers voluntarily participated in this study. The findings identified women, white-collar workers, those with longer working hours, and individuals with low exercise intentions as high-risk groups for insufficient overall PA in multiple linear regression analysis. After controlling for covariates, occupation was found to be associated with both overall PA (β = 0.146) and work-related PA (β = 0.236). Shift workers exhibited higher levels of work-related PA (β = 0.234). Furthermore, exercise intentions showed associations with overall PA (β = 0.243), leisure-time PA (β = 0.523), and transportation PA (β = 0.176) but did not demonstrate a significant relationship with work-related PA. This study highlights the importance of implementing comprehensive approaches in workplace health promotion programs aimed at enhancing various types of PA. Strategies should focus on improving exercise intentions to boost leisure-time and transportation PA, while work-related PA requires tailored interventions based on job-specific factors.

## 1. Introduction

Physical activity (PA) encompasses any body movement driven by skeletal muscles that requires energy expenditure. PA is strongly linked with a reduced risk of premature mortality and major non-communicable diseases [1]. The World Health Organization recommends that adults aged 18–64 engage in at least 150–300 min of moderate-intensity aerobic PA per week, or at least 75–150 min of vigorous-intensity aerobic PA, or an equivalent combination of both throughout the week. However, more than a quarter of the global adult population remains inadequately active [2]. In Taiwan, the situation is particularly concerning. Only 25.8% of the adult population in Taiwan manage to achieve the recommended 150 min of moderate-intensity exercise per week [3]. Despite a rise in the proportion of regular exercisers from 18.8% in 2006 to 35.0% in 2023 [3], the prevalence of insufficient PA among individuals aged 18 and above remains alarmingly high at 47.3% [4]. Among these individuals, those aged 30–54 exhibit the lowest activity levels, and they are of prime working age.

The workplace has been acknowledged as a pivotal setting for promoting PA among adults. PA promotion in the workplace holds promise as an integrated initiative that tangibly improves worker health and augments business performance [5,6]. Due to differing work characteristics, blue-collar and white-collar workers display varying levels of PA both at work and during leisure time [7]. Research also highlights that shift work significantly influence PA levels. Time spent engaged in sedentary behavior is lower in shift workers than non-shift workers [8,9]. A nationwide survey reveals that primary reasons for the lack of PA among non-exercising individuals in Taiwan include work-related fatigue (22.5%), laziness (21.4%), and lack of time due to work commitments (21.2%) [3]. However, whether these reasons constitute genuine influencing factors or are mere convenient excuses warrants further elucidation. Research on this topic remains limited in Taiwan, necessitating an exploration of the correlation between job-related factors such as occupational category, whether taking shift work, working hours, work-related burnout, and various forms of PA.

Additionally, research conducted in Taiwan indicates that gender disparities in PA emerge from childhood, with boys engaging in significantly more PA than girls, and this gap widens during adolescence [10]. As individuals enter middle age, this gender gap in PA levels changes, with men becoming less active than women [11]. These findings suggest that gender and age are key factors influencing PA levels. Moreover, although a weak association between PA and body mass index (BMI) is generally found among non-obese individuals, BMI is significantly associated with PA in obese individuals [12,13]. Given the multitude of factors that may influence individual PA levels, demographic variables such as gender, age, and BMI should be considered in studies examining PA.

PA is typically assessed across dimensions including leisure-time PA, work-related PA, transportation PA, and domestic and gardening activities [14]. In the realm of workplace health promotion, attention often centers around the former three dimensions, excluding domestic and gardening activities [9,15,16]. Measurement of PA can be categorized into objective instruments (direct measurement) and subjective questionnaires (indirect measurement), with the specific approach contingent upon particular research objectives [17]. Utilizing objective instruments in the workplace presents challenges. A review paper highlights that self-reported questionnaires predominantly serve as the method for data collection on PA and sedentary behavior at work [18]. Thus, the questionnaires used must reliably reflect energy expenditure.

Implementation intentions serve as a potent strategy for promoting health-related behaviors; exercise intentions, in particular, present a cost-effective means to promote PA and exercise [19,20]. For certain individuals, exercise intentions facilitate the translation of intentions into behavior. Within the context of PA, the Transtheoretical Model provides a framework for conceptualizing and measuring behavior change, in addition to facilitating individualized and adaptable promotion strategies [21]. This model, developed as a comprehensive model of behavior change, integrates intention, behavior, and temporal aspects into a unified approach. This model, which includes five stages, suggests that health behavior change is not an all-or-nothing phenomenon but rather a dynamic process that evolves over time. Individuals undergoing behavior change may cycle through different stages repeatedly. Among these stages, the least amount of change occurs in the precontemplation stage, while the most significant changes take place in the contemplation stage. In this stage, individuals gradually become more aware and engage in continuous self-evaluation, eventually leading them to enter the behavior change phase based on the results of their internal self-assessment [22]. The types of interventions required will thus vary depending on the stage individuals are in. When applied to exercise adoption, these stages of change encompass precontemplation (no intention to exercise), contemplation (intention to exercise within the next six months), preparation (intention to exercise within the next 30 days), action (regular exercise for less than six months), and maintenance (regular exercise for more than six months). Intention plays a pivotal role in delineating the early stages of change. However, relevant research predominantly focuses exclusively on regular exercise or leisure-time PA. Clarification concerning their influences on other types of PA is warranted. In brief, this study aims to explore the relationships between job-related factors and exercise intentions in relation to overall PA and its subdivisions.

## 2. Materials and Methods

### 2.1. Participants and Recruitment

This research adopts a cross-sectional design and adheres to the principles outlined in the Declaration of Helsinki. Approval for this study was obtained from the Institutional Review Board of Chung-Shan University Hospital in Taiwan (CSHIRB No: CS18263). Researchers visited workplaces and explained the study’s purpose to the occupational health staff. To ensure the representativeness of the sample, one out of every three employees was randomly selected based on their employee numbers. The inclusion criteria for participant recruitment were full-time workers aged 20–65 who were willing to participate. Exclusion criteria included part-time workers, illiterate individuals, and those unwilling to participate. A total of 465 full-time workers were invited to participate in the study. The researchers respected the respondents’ autonomy, ensured confidentiality, and obtained informed consent from workers willing to participate. Ultimately, 400 participants agreed to take part in the study and provided complete questionnaire responses. Using the G*Power 3.1.9.7 software to calculate sample size based on the effect size of an R-square of 0.03 with a power of 0.80, a sample size larger than 202 was enough. For additional details regarding the study procedure, please refer to our previous publication [23].

### 2.2. Measures

The research employed a questionnaire survey method for data collection, encompassing demographic characteristics, job-related factors, exercise intentions, and PA levels. The section on demographic characteristics included inquiries on gender, age, education level, marital status, and BMI. BMI was computed as weight in kilograms divided by height in meters squared (kg/m^2^), and BMI status was categorized as underweight (BMI < 18.5), ideal weight (18.5 ≤ BMI < 24.0), overweight (24.0 ≤ BMI < 27.0), and obese (BMI ≥ 27.0) following the definition provided by the Health Promotion Administration, Ministry of Health and Welfare in Taiwan [24].

#### 2.2.1. Job-Related Factors

Job-related factors comprised occupation, whether the worker engaged in shift work, working hours, and work-related burnout. Occupation was classified into two categories: white-collar worker (encompassing management, professional, technician, clerical support worker, and service worker) and blue-collar worker (including crafts worker and machine operator) [25].

#### 2.2.2. Burnout

The Copenhagen Burnout Inventory, developed by Kristensen et al. [26], was used to gauge the extent of work-related burnout among participants. This inventory is a valid and reliable questionnaire measuring personal burnout (6 items), work-related burnout (7 items), and client-related burnout (6 items). According to the theoretical framework of this inventory, these subscales can be utilized independently based on the target population. For the current study, the work-related burnout subscale was solely utilized. The internal consistency of this subscale, as measured by Cronbach’s alpha, was determined to be 0.934. Participants rated their responses on a 5-point scale, ranging from 0 (never) to 4 (always), with higher scores indicating heightened levels of burnout.

#### 2.2.3. Exercise Intentions

The levels of exercise intention were assessed utilizing the Transtheoretical Model [6], employing a 5-stage measurement to evaluate exercise behavior. These stages include precontemplation, contemplation, preparation, action, and maintenance. The Transtheoretical Model within the PA context provides a framework for both the conceptualization and measurement of behavior change with a validated construct validity [21]. Evidence suggests that adjacent stages can be effectively distinguished by intention to participate in exercise for adults, although no significant difference was found between action and maintenance [27]. Hence, exercise intentions are categorized as no (precontemplation), mild (contemplation), moderate (preparation), and strong (action and maintenance) levels for this study. Higher scores indicate a higher level of exercise intention [27].

#### 2.2.4. Level of Physical Activity

A self-reported questionnaire, comprising details about leisure-time, transportation, work-related, and overall PA, served as the data collection tool. The PA-related questions were adapted from previous studies conducted in China and in Nordic countries [28,29,30]. This instrument, previously validated, demonstrates a high correlation with physical fitness measured through energy expenditure [31,32].

Leisure-time PA was categorized as follows: (1) low: indicating almost complete inactivity or engaging in minor PA, albeit not at a moderate or high intensity; (2) moderate: involving some moderate-intensity PA for more than 4 h per week, such as walking, cycling, or light gardening; and (3) high: inclusive of vigorous PA for more than 3 h per week, such as running, jogging, skiing, swimming, or heavy gardening.

Transportation PA was categorized as follows: (1) low: using motorized transportation; (2) moderate: walking or bicycling for 1–29 min; and (3) high: walking or bicycling for 30 min or more.

Work-related PA was categorized as follows: (1) low: physically very easy work with seated office tasks (e.g., secretarial work); (2) moderate: work involving standing and walking (e.g., store assistant, light industrial work); and (3) high: work involving walking and lifting, or heavy manual labor (e.g., industrial work, farm work).

All three subdivisions of PA were consolidated and reclassified into three categories to determine the overall PA level: (1) low: participants reported low levels of leisure-time, transportation, and work-related PA; (2) moderate: participants reported moderate to high levels of PA in only one of the three subdivisions; and (3) high: participants reported moderate to high levels of PA in two or all three subdivisions.

### 2.3. Statistical Analysis

The Chi-square test was adopted to compare the differences in overall PA levels by demographic characteristics and job-related variables. Cramér’s V was employed to assess the effect size. Pearson’s correlation was used to analyze the relationships between the continuous variables. Mean, standard deviation, skewness, and kurtosis were calculated for the continuous variables to evaluate normality. The demographic characteristics showing significant differences in overall PA were controlled for in multiple linear regressions. Accordingly, gender and education level were controlled for in multiple linear regressions. The categorical variables, including gender, whether taking shift work, and occupational category, were transformed into dummy variables beforehand. Female, without shift work, and white-collar worker were coded as referents. Multiple linear regression analysis was adopted to investigate the relationships between job-related factors and PA levels, incorporating overall PA and its subdivisions. The roles of exercise intentions on PAs were explored as well. All statistical procedures were performed using SPSS-20.0 software for Microsoft Windows (IBM Corp., Armonk, NY, USA); a *p*-value less than 0.05 was considered statistically significant.

## 3. Results

Out of the 465 distributed questionnaires, 400 were successfully collected, yielding an 86.0% response rate. Among the participants, 89.8% were male, and 10.3% were female. The average age was 37.7 years (SD = 6.1). The majority of the participants were married (65.5%), held a bachelor’s degree (76.8%), identified as white-collar workers (80.8%), and did not engage in shift work (71.8%). Approximately one-third of the participants had an ideal weight (34.3%), while 31.8% were overweight, and 32.3% were classified as obese. The prevalence of overall PA levels was as follows: 5.0% for low level, 26.3% for moderate level, and 68.8% for high level. Table 1 details the overall PA levels concerning demographic characteristics and job-related variables. Gender, education level, occupational category, and shift work demonstrated significant differences in overall PA levels. 

Table 2 presents the bivariate correlations and descriptive statistics for the research variables. All skewness and kurtosis coefficients were between −1.56 and 1.20, showing that they were close to a normal distribution. Age was positively associated with working hours (r = 0.113, *p* < 0.05) and negatively associated with work-related PA (r = −0.151, *p* < 0.01). Working hours exhibited a positive association with work-related burnout (r = 0.154, *p* < 0.01) and a negative association with overall PA (r = −0.117, *p* < 0.05). Exercise intention displayed positive associations with overall PA (r = 0.211, *p* < 0.01), leisure-time PA (r = 0.529, *p* < 0.01), and transportation PA (r = 0.179, *p* < 0.01). Regarding specific PAs, overall PA showed positive associations with leisure-time PA (r = 0.471, *p* < 0.01), transportation PA (r = 0.457, *p* < 0.01), and work-related PA (r = 0.452, *p* < 0.01). Leisure-time PA also exhibited a positive relationship with transportation PA (r = 0.118, *p* < 0.05). However, work-related PA did not show significant associations with leisure-time PA or transportation PA.

Table 3 summarizes multiple linear regressions predicting PAs based on job-related factors and exercise intentions. For overall PA (F = 9.706, *p* < 0.001; adjusted R^2^ = 0.132), with gender and education level controlled, occupational category (β = 0.146, *p* < 0.01), working hours (β = −0.119, *p* < 0.05), and exercise intentions (β = 0.243, *p* < 0.001) were significant factors. The other multiple linear regressions were also controlled with covariates. For leisure-time PA (F = 24.300, *p* < 0.001; adjusted R^2^ = 0.290), only exercise intentions (β = 0.523, *p* < 0.001) demonstrated significance. Similarly, for transportation PA (F = 2.874, *p* < 0.01; adjusted R^2^ = 0.032), only exercise intentions (β = 0.176, *p* < 0.001) displayed significance. For work-related PA (F = 22.733, *p* < 0.001; adjusted R^2^ = 0.277), occupational category (β = 0.236, *p* < 0.001) and shift work (β = 0.234, *p* < 0.001) were significant.

## 4. Discussion

The aim of this study was to investigate the relationship between job-related factors and exercise intentions concerning overall PA and its three subdivisions: leisure-time PA, transportation PA, and work-related PA. The findings identify specific demographic and job-related factors, highlighting women, white-collar workers, individuals with extended working hours, and those with diminished exercise intentions as high-risk groups for inadequate overall PA. Notably, exercise intentions showed significant correlations with leisure-time PA and transportation PA. Conversely, work-related PA was associated with shift work and occupational categories. The present study emphasizes the necessity of employing tailored approaches in workplace health promotion initiatives, thereby acknowledging the diverse influences on various forms of PA.

This study demonstrates that exercise intention significantly influences overall PA, leisure-time PA, and transportation PA. Particularly, exercise intention reflects an individual’s willingness and proactive engagement, exerting a more pronounced impact on leisure-time PA. The concept of intention plays a central role in theories explaining health behaviors, including PA engagement [33,34]. Despite a considerable intention–behavior gap in PA [35], intention remains a significant correlate of PA behavior [20,36,37]. The intricate interplay between exercise intentions and various dimensions of PA underscores the importance of addressing intention as a critical factor in shaping health behaviors, especially in the context of full-time employment. Strategic interventions aimed at enhancing exercise self-esteem and alleviating exercise barriers are pivotal for fostering sustainable exercise habits among workers [23].

The observed correlation between exercise intention and transportation PA, while significant, displays a slight diminution, possibly attributed to the accessibility of public transportation and time constraints. The workplace setting of this study, located in a rural area with participants managing familial responsibilities, suggests potential limitations in enhancing transportation-related PA. Recognizing the impact of neighborhood physical environmental variables on transportation PA [38], workplace interventions, such as adjusting parking distances or transportation boarding areas, may contribute to augmenting transportation PA. Health and transportation researchers have examined PA from different perspectives for a long time. In the 2000s, they began encountering studies from the transportation field [39]. PA and active transport, usually termed “non-motorized transport”, need more collaboration. In the future, it will be necessary to explore the essential factors influencing transportation PA for workplaces with convenient public transportation in urban areas.

As expected, exercise intention was not significantly associated with work-related PA, suggesting that factors influencing PA during work hours may not solely depend on an individual’s exercise intention. Job-related variables emerged as more prominent determinants of work-related PA, with blue-collar and shift workers exhibiting significantly higher levels compared to their white-collar counterparts and non-shift workers. Previous studies show similar findings [9,16]. Even using objective measurement, workers with shift work were found to be less sedentary than those with non-shift work [8,40]. Shift work is associated with various health problems, such as cardiovascular disease and metabolic syndrome [16,41]. Shift workers, therefore, require a better healthy lifestyle and to engage in health behaviors in order to decrease health hazards. However, shift workers are found to have insufficient sleep, poor sleep quality, and poor nutritional intake [15]. A prospective cohort study confirmed that irrespective of the level of work-related PA, a consistently lower risk with increasing leisure-time PA was found for both cardiovascular and all-cause mortality among workers [42]. Fortunately, shift work was not significantly related to leisure-time PA and transportation PA in the current study. Increasing these two types of PA might, therefore, be another approach to promote shift workers’ health.

A review article found that PA levels at work are generally low, while sedentary behavior is high. This was largely a function of occupation; white-collar workers exhibited more sedentary behavior than blue-collar workers [18]. Our study shows similar findings, with white-collar workers reporting lower levels of overall PA and work-related PA. Regarding overall PA, this aligns with the extant literature, indicating a propensity for higher overall PA levels among those engaged in manual labor [43]. Other research also finds that full-day sedentarism is more likely to occur among white-collar workers and in those with higher levels of education [44,45]. Moreover, the negative association between long work hours and overall PA underscores the potential risk posed by extended working hours, particularly among white-collar workers engaged in overtime. This situation emphasizes the need for targeted interventions to address the specific challenges faced by this demographic in maintaining sufficient levels of overall PA. Additionally, although work-related fatigue and lack of time due to work commitments have been reported as reasons for Taiwanese workers not engaging in exercise [3], working hours and work-related burnout were not significantly associated with any subdivision of PA. Addressing how to debunk these misconceptions and improve workers’ intentions may present a challenge.

The present study was conducted with a rigorous approach; nonetheless, it is essential to acknowledge certain limitations. The study’s participants were exclusively sourced from the manufacturing industry, and the sample size was relatively small. This might impact the generalizability of the study findings. Additionally, the assessment of PA relied on self-reported measures, introducing the possibility of overestimation or underestimation. Moreover, the voluntary participation of workers may have led to selective bias, potentially impacting the generalizability of the study’s results. Consequently, caution is advised when extrapolating and applying the findings of this study.

## 5. Conclusions

Unlike most previous studies that focus on leisure-time PA, this study offers a comprehensive analysis of overall PA and its subdivisions. The findings reveal the complex relationships between job-related factors, exercise intentions, and various types of PA. Tailored workplace health promotion programs should address the distinct influences on leisure-time PA, transportation PA, and work-related PA. Exercise intention was the key factor significantly associated with overall, leisure-time, and transportation PA. Interestingly, long working hours and burnout were not linked to any PA subdivisions. These findings highlight the importance of enhancing workers’ exercise intentions to promote both leisure-time and transportation PA. Future research should assess the effectiveness of workplace programs in improving exercise intentions. Work-related PA was largely influenced by occupational factors, necessitating job-specific strategies. White-collar workers, who often have sedentary jobs, may benefit from structured exercise breaks and ergonomic adjustments. Meanwhile, shift workers, despite exhibiting higher levels of work-related PA, could benefit from initiatives that promote transportation and leisure-time PA, such as encouraging cycling or walking as modes of transportation or flexible exercise options like 24 h gym access or digital platforms. Tailoring interventions to specific occupational groups remains essential for improving all types of PA.

## Figures and Tables

**Table 1:** The comparisons of the level of overall physical activity by demographic variables and job-related factors.

Variables	Total (*n* = 400)	Low Level (*n* = 20, 5.0%)	Moderate Level (*n* = 105, 26.3%)	High Level (*n* = 275, 68.8%)	χ^2^	*p*	Effect Size
	*n* (%)	*n* (%) ^a^	*n* (%) ^a^	*n* (%) ^a^			
Gender					47.1	<0.001	0.343
female	41 (10.3)	0 (0.0)	29 (70.7)	12 (29.3)			
male	359 (89.8)	20 (5.6)	76 (21.2)	263 (73.3)			
Marital status					1.0	0.601	0.050
not-married	138 (34.5)	5 (3.6)	35 (25.4)	98 (71.0)			
married	262 (65.5)	15 (5.7)	70 (26.7)	177 (67.6)			
Education					12.8	0.012	0.127
senior high	63 (15.8)	4 (6.3)	10 (15.9)	49 (77.8)			
graduate	307 (76.8)	12 (3.9)	83 (27.0)	212 (69.1)			
postgraduate	30 (7.5)	4 (13.3)	12 (40.0)	14 (46.7)			
BMI					8.6	0.198	0.104
underweight	6 (1.5)	0 (0.0)	3 (50.0)	3 (50.0)			
ideal weight	136 (34.3)	6 (4.4)	46 (33.8)	84 (61.8)			
overweight	126 (31.8)	8 (6.3)	27 (21.4)	91 (72.2)			
obesity	128 (32.3)	6 (4.7)	29 (22.7)	93 (72.7)			
Occupation					14.9	0.001	0.193
white-collar	323 (80.8)	19 (5.9)	96 (29.7)	208 (64.4)			
blue-collar	77 (19.3)	1 (1.3)	9 (11.7)	67 (87.0)			
Shift work					15.8	<0.001	0.199
without	287 (71.8)	14 (4.9)	91 (31.7)	182 (63.4)			
with	113 (28.3)	6 (5.3)	14 (12.4)	93 (82.3)			

^a^ Calculated as a percentage of valid counts within each row’s cells. Effect size is Cramér’s V.

**Table 2:** The correlations of research variables.

Variables	1	2	3	4	5	6	7	8
1 Age	–							
2 Working hours	0.113 *	–						
3 Work-related burnout	−0.068	0.154 **	–					
4 Exercise intentions	−0.005	0.012	−0.052	–				
5 Overall PA	−0.067	−0.117 *	−0.045	0.211 **	–			
6 Leisure-time PA	−0.056	−0.009	−0.067	0.529 **	0.471 **	–		
7 Transportation PA	−0.014	−0.019	−0.018	0.179 **	0.457 **	0.118 *	–	
8 Work-related PA	−0.151 **	−0.063	−0.005	−0.085	0.452 **	0.003	−0.007	–
Mean	37.72	44.00	41.16	2.65	2.64	1.75	1.94	1.74
Standard deviation	6.14	4.27	14.34	0.96	0.58	0.89	0.59	0.70
Skewness	−0.16	0.73	0.19	−0.19	−1.34	0.51	0.02	0.41
Kurtosis	−0.04	1.20	0.60	−0.90	0.81	−1.56	−0.17	−0.93

* *p* < 0.05; ** *p* < 0.01; standard error of skewness is 0.122, and the standard error of kurtosis is 0.243.

**Table 3:** Summary of multiple linear regression predicting overall PA and its subdivisions.

Variables	Overall PA				Leisure-Time PA				Transportation PA				Work-Related PA			
	B	SE	*β*		B	SE	*β*		B	SE	*β*		B	SE	*β*	
Constant	2.597	0.378			0.059	0.531			2.286	0.412			1.098	0.423		
Gender	0.334	0.093	0.176	***	0.401	0.131	0.136	**	−0.115	0.101	−0.059		0.563	0.104	0.243	***
Education	−0.084	0.059	−0.069		0.148	0.083	0.079		−0.111	0.064	−0.089		−0.157	0.066	−0.106	*
Occupation	0.212	0.072	0.146	**	0.074	0.101	0.033		0.072	0.078	0.048		0.419	0.080	0.236	***
Shift work	0.083	0.065	0.065		−0.057	0.092	−0.029		−0.115	0.071	−0.087		0.364	0.073	0.234	***
Working hours	−0.016	0.007	−0.119	*	−0.007	0.009	−0.033		−0.003	0.007	−0.022		−0.006	0.007	−0.039	
Work-related burnout	−0.001	0.002	−0.033		−0.002	0.003	−0.040		0.000	0.002	−0.008		−0.001	0.002	−0.027	
Exercise intentions	0.146	0.028	0.243	***	0.489	0.040	0.523	***	0.109	0.031	0.176	***	−0.013	0.032	−0.017	
Adjusted R^2^	0.132				0.290				0.032				0.277			
F	9.706			***	24.300			***	2.874			**	22.733			***

Β denotes unstandardized regression coefficient; SE denotes standard error; β denotes standardized regression coefficient. Female, white-collar worker, and without shift work were coded as referents. * *p* < 0.05; ** *p* < 0.01; *** *p* < 0.001.

## Data Availability

The research data will be available upon request to the corresponding author.
